# Prediction of Molecular Physicochemical Properties Using Molecular Structure and Characteristics

**DOI:** 10.3390/molecules31213986

**Published:** 2026-11-05

**Authors:** Jiahuan Jin, Jie Li

**Affiliations:** School of Computer Science and Technology, Harbin Institute of Technology, Harbin 150001, China; 20b903085@stu.hit.edu.cn

**Keywords:** molecular physicochemical property prediction, motif structure, augmented graph, characteristics optimized selection

## Abstract

The prediction of molecular physicochemical properties—such as solubility, hydration free energy, and lipophilicity—plays a crucial role in multiple fields, including drug discovery and materials science. Current prediction methods predominantly fall into two categories: machine learning methods based on molecular descriptors and deep learning methods that learn representations directly from molecular structures. However, when evaluated under rigorous scaffold splitting, these methods often exhibit limited generalization capability, resulting in unsatisfactory prediction accuracy. In this study, a method named PMPP is proposed to predict the physicochemical properties of molecules, which effectively utilizes enhanced graph representations that capture molecular motif structures and optimized selection of pharmacophore and physicochemical characteristics. The framework of PMPP contains three parts: molecular structure representation extraction, molecular characteristics extraction and optimized selection, and molecular physicochemical property prediction model construction. Experimental results demonstrate that PMPP achieves superior predictive accuracy, with lower Root Mean Square Error (RMSE) scores on three benchmark datasets: 0.787 (ESOL), 1.875 (FreeSolv), and 0.615 (Lipophilicity). These results outperform six state-of-the-art methods, including GROVER, MGSSL, MolCLR, KANO, HiMol and Mol-AE. Furthermore, our analysis reveals that robust prediction relies not only on molecular graph and motif representations but also on key pharmacophore and physicochemical characteristics—such as hydrophobic group count and LogP—which show strong correlations with the target molecular properties.

## 1. Introduction

The physicochemical properties of molecules significantly influence drug absorption, metabolism, and excretion, making them critical factors in pharmacokinetic and ADMET (Absorption, Distribution, Metabolism, Excretion, and Toxicity) analysis with broad implications across multiple fields [1,2,3]. For instance, the solubility property of a drug molecule determines the proportion of its active substance absorbable in the gastrointestinal tract [4]. Drug development is a costly and risky process, with 90% of drug candidates failing during clinical phases due to the high cost of experimental trials and inadequate biomedical properties [5]. Therefore, accurate prediction of molecular physicochemical properties can assist drug developers in compound screening and determining appropriate dosing regimens.

In order to predict the physicochemical properties of drug molecules, researchers have developed several methods, which can be divided into four categories according to the information of the input molecule: sequence-based [6,7], molecular graph-based [8,9], motif-based [10,11,12], and hybrid input-based [13,14] methods. Sequence-based methods utilize natural language processing techniques to represent molecular sequences (e.g., SMILES or SELFIES [15]) for molecular property prediction. Graph-based methods construct molecular graphs by treating atoms as nodes and chemical bonds as edges and employ graph neural network models to learn the representation of molecular graphs, thereby predicting molecular properties. MolCLR [8], for example, predicts molecular properties through contrastive learning on molecular graphs. However, both sequence-based and graph-based methods overlook distinctive chemical structures such as rings and functional groups, which play pivotal roles in determining chemical properties. Motif-based methods, such as MGSSL [10] and HiMol [11], obtain hierarchical molecular representations by constructing molecular motifs to enhance the accuracy of molecular property prediction. In recent years, to improve the prediction performance of molecular physicochemical properties, researchers have proposed hybrid input-based methods [13,14]. These methods refine motif-based methods by incorporating additional information. For example, KANO [13] adds chemical element knowledge graph information on the basis of motif information to extract potential inter-atomic relationships. However, when evaluated under rigorous scaffold splitting, these molecular property prediction methods often exhibit limited generalization capability, resulting in unsatisfactory prediction accuracy [13].

Critically, molecular physicochemical property prediction requires focusing not only on molecular graphs and motifs but also on characteristics like the octanol–water partition coefficient (LogP) and hydrophobic group count because this information has an obvious correlation with the physicochemical properties of molecules [16,17]. However, existing methods do not take advantage of structural, fingerprint and characteristics information. While machine learning has emerged as a powerful tool for such predictions, models often suffer from poor generalization when evaluated on compounds with novel molecular scaffolds, limiting their utility in real-world virtual screening. Therefore, in this study, we propose a hybrid deep learning Prediction of Molecular Physicochemical Property (PMPP) framework that integrates essential physicochemical descriptors—specifically, hydrogen bond donors, molecular weight, and the number of hydrophobic groups—as prior knowledge features to guide model training. On the basis of the existing graph structure features, further extraction and optimization of molecular features can better improve our understanding of molecular physicochemical properties. We experimentally verify the performance of PMPP on three widely used molecule property datasets, and the results show that our method is competitive.

## 2. Results

### 2.1. Predictive Performance Evaluation of PMPP

The three widely used molecule property datasets, ESOL [18], FreeSolv [19], and Lipophilicity [20], are used to predict molecular solubility, hydration free energy, and lipophilicity, respectively. Table 1 reports the average Root Mean Square Error (RMSE) values of PMPP across five independent runs on the test sets partitioned by molecular scaffold across three benchmark datasets, yielding 0.772 (ESOL), 1.874 (FreeSolv), and 0.653 (Lipophilicity). For the training and validation sets, the best RMSE values are 0.459 and 0.789 for ESOL, 0.552 and 3.087 for FreeSolv, and 0.377 and 0.568 for Lipophilicity, respectively. Different studies adopt distinct parameter optimization schemes [8,9,10,11,12,13], including fixed training epochs, early stopping mechanisms with varied monitoring metrics and thresholds, and multi-stage training paradigms to improve model performance on the test set [21,22,23,24,25]. High performance on the training or validation set may merely arise from overfitting and cannot reliably reflect the true predictive capacity of a model [26]. Hence, performance on an independent test set is widely recognized as the primary benchmark for comparing model generalization across different studies [8,9,10,11,12,13]. To evaluate the effectiveness of the proposed method, we compared PMPP against several state-of-the-art methods—namely, GROVER [12], MGSSL [10], MolCLR [8], KANO [13], HiMol [11], and Mol-AE [9]—on the same three datasets for molecular physicochemical property prediction. The corresponding test RMSE results are also summarized in Table 1. The comparative analysis shows that PMPP consistently outperforms all six competing methods across the evaluated benchmarks.

### 2.2. Ablation Experiments

In order to obtain a deeper insight into the importance of each module in PMPP, an ablation experiment was designed from various perspectives.

In the first perspective, only the molecular structure representation module is employed (PMPP-w-S). In the second perspective, only the molecular characteristics representation module is utilized, containing PMPP-w-CS, PMPP-w-CI, and PMPP-w-CO, respectively. For PMPP-w-CS, all characteristics are standardized, without any additional optimized selection. For PMPP-w-CI, all characteristics are processed according to the molecular characteristics extraction pipeline described in the Section 4.2.2. For PMPP-w-CO, all characteristics are initially processed the same as the PMPP-w-CI variant and subsequently optimized and selected by a Pearson correlation-based method. In the third perspective, molecular property prediction is performed using fixed-length structural fingerprints, namely, MACCS (PMPP-w-MACCS) and Morgan (PMPP-w-Morgan), with 166- and 881-dimensional features, respectively. The test RMSE and Pearson correlation coefficient (PCC) results of the ablation studies on the three datasets partitioned by molecular scaffold are shown in Table 2.

Across the three datasets, PMPP-w-CO consistently yielded lower RMSE values (0.807, 1.792, and 0.755) compared to those of PMPP-w-CI (0.902, 1.900, and 0.854) and PMPP-w-CS (1.255, 2.820, and 0.791). In contrast to the structure-based method (PMPP-w-S), the fingerprint-based methods, PMPP-w-MACCS and PMPP-w-Morgan, yielded considerably higher RMSE values across all datasets. As shown in Table 2, molecular characteristics information and its optimized selection play a crucial role in improving the predictive performance of the graph structure-based physicochemical property model on the scaffold-split test dataset.

### 2.3. Results of the Importance of Molecular Characteristics Representation

**Figure 1:**
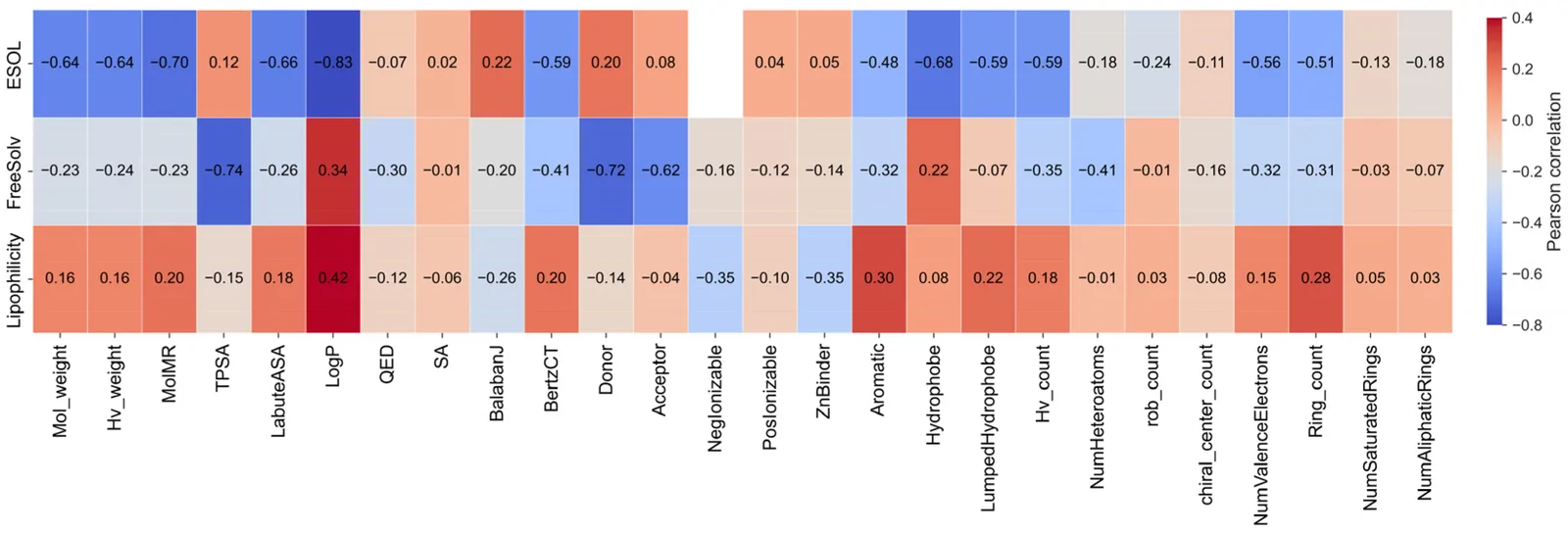
The heatmap of Pearson correlations between the 26 characteristics and the physicochemical properties in three datasets.

The Pearson correlations between the 26 molecular characteristics and the physicochemical properties of all molecules in the three datasets are shown in Figure 1. It can be seen that there is a certain relationship between molecular characteristics and physicochemical properties. In the ESOL dataset, the top four absolute Pearson correlation characteristics for solubility prediction are LogP (0.83), Wildman–Crippen MR value (0.70), the number of hydrophobic atoms (0.68), and Labute’s Approximate Surface Area (0.66). In the FreeSolv dataset, the top four absolute Pearson correlation characteristics for hydration free energy prediction are topological polar surface area (0.74), donor (0.72), acceptor (0.62), and the number of hetero atoms (0.41). In the Lipophilicity dataset, the top four absolute Pearson correlation characteristics for lipophilicity prediction are LogP (0.42), moieties possessing negative ionizability (0.35), moieties associated with Zn ion binding (0.35), and the number of aromatic rings (0.30).

Directly employing these molecular characteristics as molecular physicochemical property predictors is prone to overfitting, as their predictive capability is largely constrained by the scale and diversity of the training data. Nevertheless, these molecular characteristics can serve as valuable prior knowledge to enhance the predictive performance of molecular graph structure-based representation learning for physicochemical properties. Particularly on the scaffold-split test set, where structural generalization remains a critical challenge, incorporating these characteristics as auxiliary information effectively improves model performance.

To evaluate the benefit of additional molecular characteristics, Table 3 lists the top five compounds from the ESOL dataset with the greatest prediction error reduction from the structure-only baseline (PMPP-w-S) to the PMPP method. For each compound, key physicochemical properties—including LogP value, number of hydrophobic groups, and aromatic ring count—are also listed. Comparative analysis reveals that the molecules that were severely overestimated or underestimated by the structure-based baseline were effectively corrected upon integration of these molecular characteristics.

### 2.4. Correlation Analysis

#### 2.4.1. Molecular Characteristics Analysis

**Figure 2:**
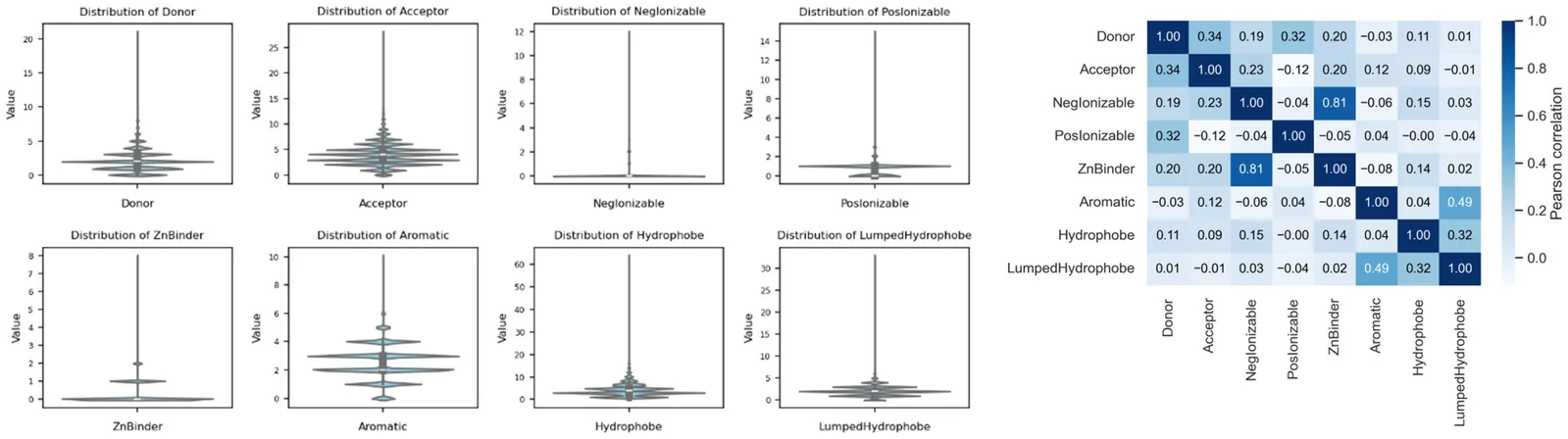
Distribution and Pearson correlations of eight pharmacophore characteristics in GuacaMol.

To systematically investigate the relationship between molecular pharmacophoric characteristics and physicochemical properties, we employed the GuacaMol benchmark suite, which was constructed from the ChEMBL 24 public database and comprises 1,591,378 unique molecular structures. For each molecule, we extracted eight core pharmacophoric characteristics—including hydrogen bond donors (HBDs), hydrogen bond acceptors (HBAs), positively ionizable moieties (PIMs), negatively ionizable moieties (NIMs), zinc-binding groups (ZBGs), aromatic rings (ARs), hydrophobic atoms (HAs), and hydrophobic groups (HGs)—along with 27 sub-classes (e.g., aromatic ring size classification: five-membered, six-membered, etc.) using the RDKit cheminformatics toolkit [27]. The frequency distributions of these pharmacophoric characteristics across the GuacaMol dataset are presented in Figure 2, revealing that the majority of drug-like molecules contain 1–3 HBDs and 2–5 HBAs, consistent with Lipinski’s Rule of Five characteristics.

Furthermore, to further dissect the inter-relationships among pharmacophoric characteristics, we computed their pairwise correlation matrix (Figure 2). The most striking association was observed between NIM and ZnBinder, with a correlation coefficient of 0.81. Within the ZnBinder sub-classes, ZnBinder5 (SMARTS: [6]-C(=O)-[O;D1], corresponding to the carboxylate moiety CC(=O)O) exhibited the strongest correlation with NIM (r = 0.90), indicating that negatively charged carboxylate groups are the predominant metal-binding pharmacophore in drug-like molecules. In contrast, the positively ionizable (PIM) feature displayed a weak negative correlation with the metal acceptor (r = −0.12). However, PIM showed a strong positive correlation with the BasicGroup sub-class (r = 0.80), defined as a set of basic nitrogen-containing moieties. Additionally, a moderate positive correlation (r = 0.32) was observed between PIM and the metal donor feature, suggesting that basic groups can serve as both hydrogen bond donors and metal-coordinating ligands in certain contexts. Regarding aromatic ring features, most aromatic rings in the GuacaMol dataset were five- or six-membered rings. A weak-to-moderate positive correlation (r = 0.49) was observed between aromatic rings and hydrophobic features. Notably, among hydrophobic sub-classes, RH6_6 (a six-membered hydrophobic ring) was the most prevalent, accounting for approximately 90% of all hydrophobic motifs, and exhibited a higher correlation with six-membered aromatic rings (r = 0.60), reflecting the ubiquitous presence of phenyl and cyclohexyl scaffolds in drug molecules.

**Figure 3:**
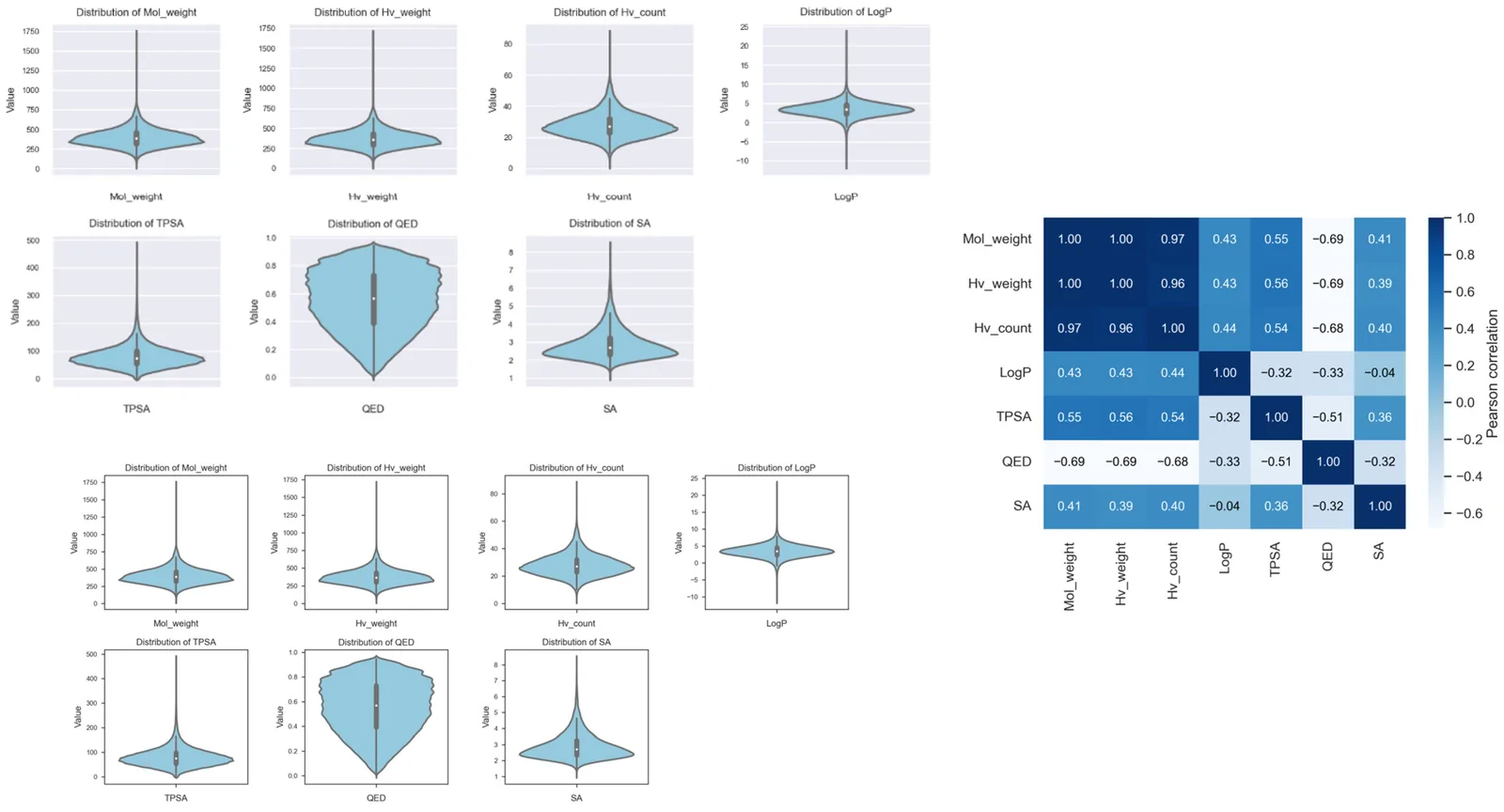
Distribution and Pearson correlations of 7 characteristics in GuacaMol.

To further contextualize our molecular property predictions, we examined the inter-relationships among seven established drug-likeness indicators: molecular weight (Mol_weight), heavy atom weight (Hv_weight), the number of heavy atoms (Hv_count), LogP, topological polar surface area (TPSA), synthetic accessibility (SA), and quantitative estimate of drug-likeness (QED). Mol_weight is a fundamental physical descriptor influencing molecular size and steric demand; Hv_weight and Hv_count are highly correlated with Mol_weight; LogP quantifies membrane partitioning propensity; TPSA reflects hydrogen-bonding capacity and polar surface exposure; SA estimates the ease of chemical synthesis; and QED is a composite score (ranging from 0 to 1) that integrates multiple drug-like properties into a single metric, with higher values indicating greater conformity to established drug profiles. Understanding the interplay among these descriptors is essential, as drug discovery inherently involves optimizing these often-competing parameters to achieve a balanced physicochemical profile. The correlation matrix (Figure 3) reveals a coherent and physically interpretable network of relationships. Mol_weight exhibited positive correlations with LogP (r = 0.43), TPSA (r = 0.55), and SA (r = 0.41) but a negative correlation with QED (r = −0.69). This pattern indicates that larger molecules tend to be more lipophilic, more polar, and more synthetically complex—yet less drug-like by QED standards. LogP showed negative correlations with TPSA (r = −0.32) and QED (r = −0.33), suggesting that increased lipophilicity is generally accompanied by reduced polar surface area and decreased drug-likeness. TPSA was negatively correlated with QED (r = −0.51) but positively correlated with SA (r = 0.36), indicating that molecules with high polar surface area tend to be synthetically more challenging and less drug-like.

**Figure 4:**
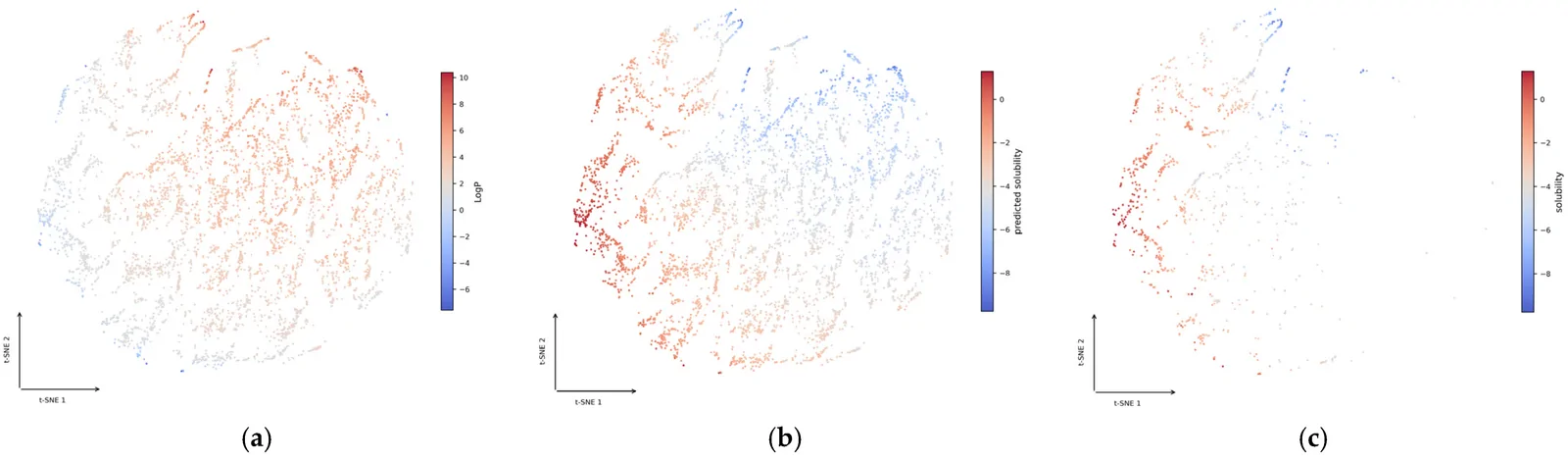
Visualization of molecular structure–characteristics representations learned by PMPP via t-SNE. (**a**) The colors of the nodes correspond to LogP values. (**b**) The colors of the nodes correspond to the predicted solubility values. (**c**) The colors of the nodes correspond to the ground-truth solubility values of molecules.

To further reveal the correlation between molecular characteristics and chemical space, we combined the ESOL, FreeSolv, and Lipophilicity datasets and used the molecular solubility prediction model trained on the ESOL dataset to predict the solubility of the molecules. During this process, the molecular structure and characteristics representations learned from the model are concatenated and mapped into a two-dimensional space using t-SNE for visualization (Figure 4). As shown in Figure 4, each node represents a molecule, and the color of the node corresponds to LogP, predicted solubility, and the ground-truth solubility (only 1128 molecules provided by the ESOL database) values in (a), (b), and (c), respectively. It is well known that there is a significant negative correlation between a compound’s LogP and solubility. This trend is consistent with the node color distribution in Figure 4a,c. By examining Figure 4b,c, it can be found that the predicted solubility and ground-truth solubility are consistent, demonstrating the satisfactory predictive capability of our model. Additionally, as shown in Figure 4b, the predicted solubility presents a significant regional distribution, indicating that the model can effectively capture the structural and characteristic similarities related to the physicochemical properties of molecules.

Furthermore, the concatenated representations of the structure and characteristics of 4200 molecules in the Lipophilicity dataset are mapped to a two-dimensional space by t-SNE (Figure S1). As shown in Figure S1, the lipophilicity of the molecules is significantly distributed in different regions, indicating that the model can effectively learn the intrinsic correlation between the lipophilicity property of molecules and their structure and characteristics.

#### 2.4.2. Molecular Structure Characteristics Analysis

To systematically investigate the relationship between molecular structural motifs and functional properties, we computed the pairwise Pearson correlation matrices between two widely used molecular fingerprint representations—MACCS keys (166 dimensions) and Morgan fingerprints (with a radius of two and 881 dimensions)—and both the pharmacophoric characteristics (eight core features) and LogP for all 1,591,378 molecules in the GuacaMol dataset. The Pearson correlations of each dimension of the MACCS and Morgan fingerprints with LogP are shown in Figures S2 and S3, respectively. Each dimension of the MACCS and Morgan fingerprints with LogP in the GuacaMol dataset are provided in Tables S2 and S3, respectively. The description of each dim of MACCS fingerprints is included in Table S2_Description. The description of each non-zero value in Morgan fingerprints is provided in Table S4. The resulting correlation heatmaps revealed that specific structural motifs exhibit remarkably strong correlations with particular pharmacophoric characteristics and target properties.

Among all fingerprint–property correlations, MACCS bit 145 (indicating the presence of more than one six-membered ring) showed the strongest positive correlation with LogP among all evaluated bits. This is consistent with the well-established medicinal chemistry principle that increased aromaticity and ring count contribute to molecular hydrophobicity—a major driving force for membrane permeability and lipophilicity.

Overall, these analyses uncover a three-way interplay between molecular structure, molecular characteristics, and physicochemical properties.

## 3. Discussion

Motivated by these results, we argue that a comprehensive molecular representation—one that integrates topological structure, pharmacophoric characteristics, and physicochemical descriptors (for property priors)—is essential for achieving high-accuracy and generalizable predictions. Accordingly, our proposed framework concatenates these complementary information sources into a unified embedding space.

We observed several noteworthy phenomena. First, by comparing the experimental ablation results of the PMPP-w-CS, PMPP-w-CI, and PMPP-w-CO methods across the three datasets presented in Table 2, it is evident that the selection and optimization of molecular characteristics can effectively improve molecular physicochemical property prediction accuracy. Furthermore, by comparing the structure-based molecular fingerprint methods (PMPP-w-MACCS and PMPP-w-Morgan) with the molecular graph-based approach (PMPP-w-S), it is evident that the graph-based method achieves superior performance in predicting molecular physicochemical properties. This advantage likely stems from the ability of graph neural networks to learn more expressive structural features through graph convolution operations. In contrast, structure-based molecular fingerprints are fixed-dimensional representations, where only a subset of dimensions may be relevant to the target properties, limiting their capacity to capture the holistic structural information of molecules. Finally, we found that PMPP surpasses its variants on scaffold-split test sets, and characteristics representation plays an important role, followed by structure representation.

## 4. Materials and Methods

### 4.1. Dataset

In order to obtain molecular structure representations, we sampled 249,456 unlabeled molecules from the ZINC15 database [28] to pre-train the GIN module. To evaluate the effectiveness of the proposed PMPP method, we compared it against other competitive methods on three popular publicly available datasets, namely, ESOL [18], FreeSolv [19] and Lipophilicity [20], with 1128, 642, and 4200 molecules, respectively. These three datasets are usually used to predict molecular solubility, hydration free energy, and lipophilicity, respectively. ESOL contains water solubility data (log solubility in mols per liter) for common organic small molecules. FreeSolv provides experimental and calculated hydration free energies for small molecules in water. Lipophilicity includes experimental octanol/water distribution coefficients (logD at pH 7.4) for 4200 compounds. These three datasets were split into training/validation/test sets using scaffold splitting with an 8:1:1 ratio. The scaffold-split method is based on the skeleton structure, ensuring that the difference in skeletons between the training and test sets is maximized.

### 4.2. PMPP Method

**Figure 5:**
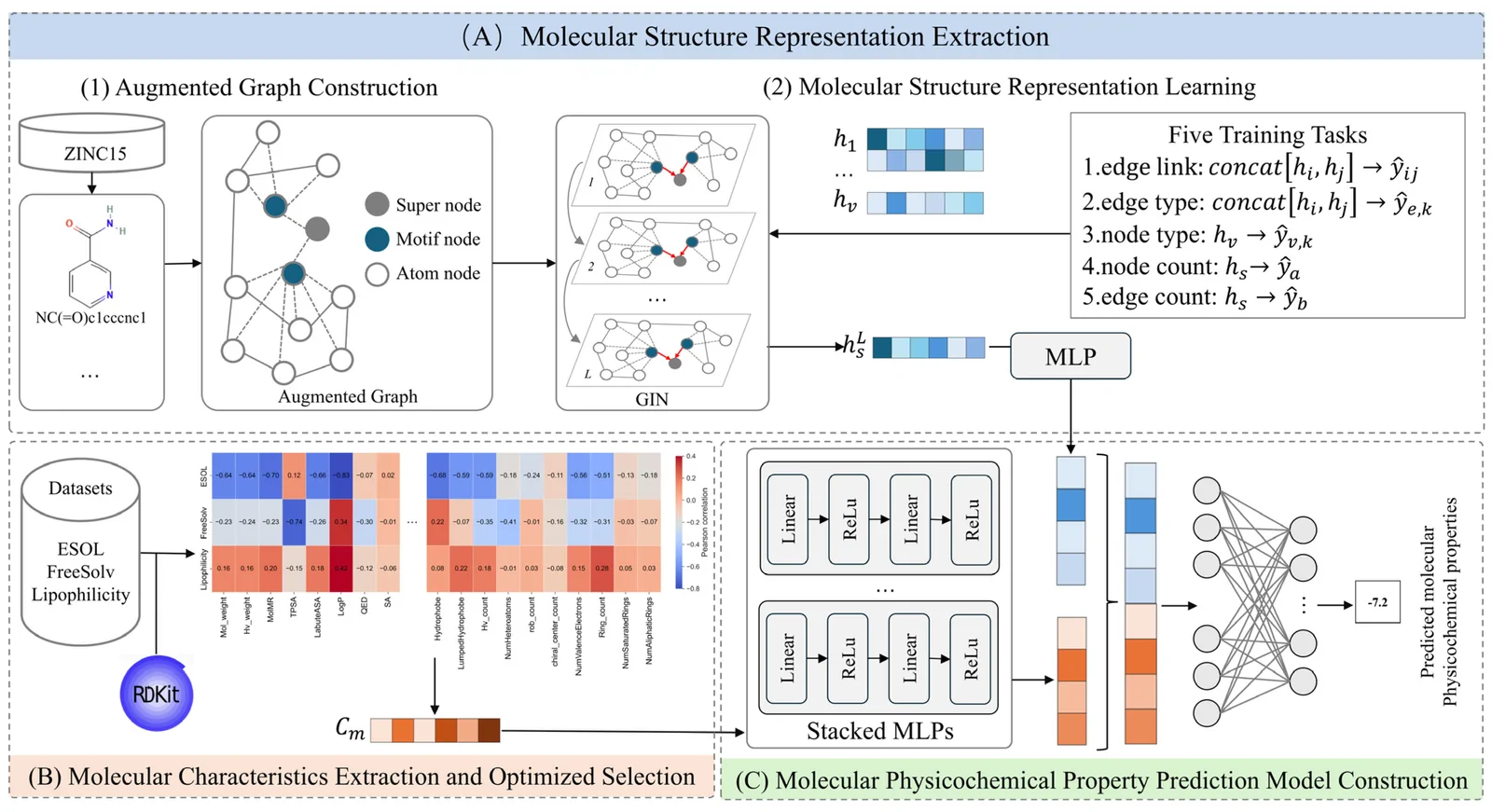
The framework of PMPP. (**A**) Molecular structure representation extraction. (**B**) Molecular characteristics extraction and optimized selection. (**C**) Molecular physicochemical property prediction model construction.

The framework of the PMPP method is shown in Figure 5. The process of molecular physicochemical property prediction contains three parts: molecular structure representation extraction, molecular characteristics extraction and optimized selection, and molecular physicochemical property prediction model construction.

#### 4.2.1. Molecular Structure Representation Extraction

- Augmented Graph Construction.

The molecular augmented graph consists of the original molecular graph, molecular motif nodes, a super node and connected edges.

First, each molecule is represented as an original molecular graph G=(V,E), where all atoms are represented as the set of nodes *V* and all bonds are represented as the set of edges *E*. Then, each molecule is divided into a series of motifs. These motifs are added to the original molecular graph as the set of motif nodes $V_{m}$, and the set of atom–motif edges $E_{am}$ is added between each motif and the atoms it covers. In our study, a motif extraction method similar to HiMol is adopted [11]. Specifically, each molecule is fragmented into small motif structures according to two rules: the BRICS rule [29] and a rule that decomposes more than one ring structure into the smallest ring structures. Subsequently, a super node $V_{s}$ is incorporated into the augmented graph to represent the entire molecule. The super node is connected to all motif nodes in the augmented graph, forming motif-super edges $E_{ms}$. The augmented graph is finally denoted by $\tilde{G}=\left(\tilde{V},\tilde{E}\right)$, containing a node set $\tilde{V}=\left[V,V_{m},V_{s}\right]$ and an edge set $\tilde{E}=\left[E,E_{am},E_{ms}\right]$.

- Molecular Structure Representation Learning.

Molecular structure representation was learned through five self-supervised training tasks using Graph Isomorphism Network (GIN) [30] on its augmented graph.

The aggregation operation of the *l*-th layer in GIN is given as follows:

(1) $$h_{v}^{l}={MLP}_{e}^{l}\left(h_{v}^{l-1}+\sum_{u\in N\left(v\right)}\left(h_{v}^{l-1}+{MLP}_{n}^{l}\left(X_{uv}\right)\right)\right),$$

where $X_{uv}$ represents the features of edge uv, where the initial edge features contain information about the edge type and whether is in a ring; $N\left(v\right)$ represents the set of neighborhoods of node *v*; $h_{v}^{l-1}$ denotes the features of node *v*, where the initial node features contain information about the node type and node degree; and ${MLP}_{n}^{l}\;and\;{MLP}_{e}^{l}$ represent multi-layer perception (MLP) for node and edge feature transitions at the *l*-th layer, respectively. After *L* iterations, the learned representation $h_{s}^{L}$ of the super node $V_{s}$ is directly used as the molecular structural representation without the need for readout operation in GIN.

The five self-supervised training tasks include: edge link prediction, edge type prediction, node type prediction, node count prediction, and edge count prediction.

For the edge link prediction task, we concatenated the two node representations $h_{i}\;and\;h_{j}$ and predicted the edge link probability ${\hat{y}}_{ij}$ through the MLP mapping function ${MLP}_{link}$, as described below:

(2) $${\hat{y}}_{ij}={MLP}_{link}\left\{concat\left[h_{i},h_{j}\right]\right\}.$$

The cross-entropy loss $L_{link}$ for this task is defined in (3):

(3) $$L_{link}=-\sum_{v_{i},v_{j}\in \tilde{V}}y_{ij}\log{\hat{y}}_{ij}+\left(1-y_{ij}\right)\log\left(1-{\hat{y}}_{ij}\right),$$

where $y_{ij}$ represent the true link between two nodes $v_{i}\;\mathrm{and}\;v_{j}$.

For the edge type prediction task, we concatenated the two node representations $h_{i}\;\mathrm{and}\;h_{j}$ and predicted the probability ${\hat{y}}_{e,k}$ that the type of an edge *e* is type *k* through the MLP mapping function ${MLP}_{edge\_type}$, as described in the following formula:

(4) $${\hat{y}}_{e,k}={MLP}_{edge\_type}\left\{concat\left[h_{i},h_{j}\right]\right\}.$$

The corresponding cross-entropy loss $L_{edge\_type}$ is defined in (5):

(5) $$L_{edge\_type}=-\frac{1}{\left|\tilde{E}\right|}\sum_{e\in \tilde{E}}\sum_{k=1}^{K_{edge}}y_{e,k}\log{\hat{y}}_{e,k},$$

where $\left|\tilde{E}\right|$ denotes the number of edges, $K_{edge}$ represents the number of edge types, and $y_{e,k}=1$ represent that an edge *e* is type *k*; otherwise, $y_{e,k}=0$.

For the node type prediction task, the input node representation $h_{v}$ is mapped through the MLP mapping function ${MLP}_{node\_type}$ to predict the node type probability ${\hat{y}}_{v,k}$, where ${\hat{y}}_{v,k}$ denotes the probability of node *v* belonging to type *k*, as described below:

(6) $${\hat{y}}_{v,k}={MLP}_{node\_type}\left\{h_{v}\right\},$$

The corresponding loss function $L_{node\_type}$ is defined in (7):

(7) $$L_{node\_type}=-\frac{1}{\left|\tilde{V}\right|}\sum_{v\in \tilde{V}}\sum_{k=1}^{K_{node}}y_{v,k}\log{\hat{y}}_{v,k},$$

where $K_{node}$ denotes the number of node types, $\left|\tilde{V}\right|$ represents the number of nodes, and $y_{v,k}$ represents the true probability that the type of a node *v* is type *k*.

Similarly, the node count probability ${\hat{y}}_{a}$ and edge count probability ${\hat{y}}_{a}$ are predicted by (8) and (9) and are obtained by processing the super node representation $h_{s}$ through two mapping functions, ${MLP}_{node\_count}$ and ${MLP}_{edge\_count}$, respectively. The corresponding SmoothL1 loss functions are defined in (9) and (11), respectively.

(8) $${\hat{y}}_{a}={MLP}_{node\_count}\left\{h_{s}\right\},$$

(9) $$L_{node\_count}=\left\{\begin{array}{c} 0.5\times {\left(y_{a}-{\hat{y}}_{a}\right)}^{2},\;if\left|\left(y_{a}-{\hat{y}}_{a}\right)\right|<1\\ \left|\left(y_{a}-{\hat{y}}_{a}\right)\right|-0.5,\;if\left|\left(y_{a}-{\hat{y}}_{a}\right)\right|\geq 1 \end{array}\right.,$$

(10) $${\hat{y}}_{b}={MLP}_{edge\_count}\left\{h_{s}\right\},$$

(11) $$L_{edge\_count}=\left\{\begin{array}{c} 0.5\times {\left(y_{b}-{\hat{y}}_{b}\right)}^{2},\;if\left|\left(y_{b}-{\hat{y}}_{b}\right)\right|<1\\ \left|\left(y_{b}-{\hat{y}}_{b}\right)\right|-0.5,\;if\left|\left(y_{b}-{\hat{y}}_{b}\right)\right|\geq 1 \end{array}\right.,$$

where $y_{a}$ and $y_{b}$ are the true node count and edge count, respectively.

The five self-supervised training tasks are weighted by a learnable weight vector $\alpha =\left[\alpha_{1},\alpha_{2},\alpha_{3},\alpha_{4},\alpha_{5}\right]$, and the overall loss function is defined as follows:

(12) $$L=\alpha_{1}L_{link}+\alpha_{2}L_{edge\_type}+\alpha_{3}L_{node\_type}+\alpha_{4}L_{node\_count}+\alpha_{5}L_{edge\_count}.$$

#### 4.2.2. Molecular Characteristics Extraction and Optimized Selection

The molecular characteristics were extracted using the RDKit tool and contain information related to the physicochemical properties of the molecules, which are listed in Table S5.

Let $C_{m}=\left\{c_{1},c_{2},\ldots c_{m}\right\}$ represent molecular characteristics, where *m* represents the number of characteristics. Eight pharmacophore characteristics were first extracted for all molecules using RDKit, including hydrogen bond donors and acceptors, moieties possessing positive or negative ionizability, and those associated with Zn ion binding, aromatic rings, hydrophobic atoms, and hydrophobic groups. Subsequently, additional intrinsic characteristics related to molecular physicochemical properties were calculated using RDKit, including molecular weight, heavy atom molecular weight, Wildman–Crippen MR value, LogP, topological polar surface area, Labute’s Approximate Surface Area, quantitative estimate of drug-likeness, synthetic accessibility score, Balaban’s J value, BertzCT value, number of heavy atoms, number of hetero atoms, number of valence electrons, number of rotatable bonds, number of chiral centers, number of smallest set of smallest rings, number of saturated rings, and number of aliphatic rings. All molecular characteristics for the ESOL, FreeSolv, and Lipophilicity datasets are provided in Table S5.

These characteristics were first divided into continuous and discrete according to their numerical values. Due to the different distribution scales of these characteristics, the continuous characteristics were independently transformed via both standardized and uniform bins, and both transformed representations were concatenated. The standardized formula is as follows:

(13) $$\bar{c_{m}}=\frac{C_{m}-\mu }{\sigma },$$

where *μ* and *σ* represent the mean and variance of a molecular characteristic $c_{m}$, respectively. Uniform binning means dividing each consecutive characteristic into *n* bins of equal width, each of which is encoded as an integer. The discrete characteristics underwent one-hot encoding. All processed characteristics were then concatenated to form the representation $C_{I}$.

In addition, the molecular characteristics were optimized and selected by a Pearson correlation-based method. Based on a statistical analysis of the Pearson correlation between these characteristics and the molecular physicochemical property labels in the training set, some characteristics with a larger absolute correlation threshold *θ* were selected as molecular characteristics $C_{o}$. This characteristics representation was subsequently fed into stacked MLP layers to generate the predicted molecular properties. The stacked MLP layers were optimized by minimizing the MSE loss between the predicted values and the ground-truth labels. The characteristics with the best performance were selected as the final optimized selected characteristics $C_{o}$.

#### 4.2.3. Molecular Physicochemical Property Prediction Model Construction

The molecular physicochemical property prediction model contains three parts.

Firstly, the molecular structure representation $h_{s}^{L}$ is dimensionally reduced through an MLP mapping function ${MLP}_{structure}$, enabling its integration with low-dimensional molecular characteristics. The formula is as follows:

(14) $${\hat{p}}^{s}={MLP}_{structure}\left\{h_{s}^{L}\right\}.$$

Subsequently, the optimized selected molecular characteristics $C_{o}$ are processed through the stacked MLP layers ${MLP}_{characteristics}$, which are designed to handle various linear combination scenarios. The formula is as follows:

(15) $${\hat{p}}^{c}={MLP}_{characteristics}\left\{C_{o}\right\}.$$

Finally, the processed molecular characteristics ${\hat{p}}^{c}$ and molecular structure representations ${\hat{p}}^{s}$ are concatenated and input into another MLP mapping function ${MLP}_{fusion}$ to predict the physicochemical properties of the molecules.

(16) $${\hat{p}}^{p}={MLP}_{fusion}\left\{concat\left[{\hat{p}}^{s},{\hat{p}}^{c}\right]\right\}.$$

The molecular physicochemical property prediction regression task is to optimize the model by computing the MSE loss between the predicted and the true molecular physicochemical properties, which contains the molecular structural loss and molecular characteristics loss as follows:

(17) $$L_{structure}=\frac{1}{N}\sum_{i=1}^{N}{\left|{\hat{p}}^{s}-p^{p}\right|}^{2},$$

(18) $$L_{characteristics}=\frac{1}{N}\sum_{i=1}^{N}{\left|{\hat{p}}^{c}-p^{p}\right|}^{2},$$

(19) $$L_{all}=\beta_{1}L_{structure}+{\beta_{2}L}_{characteristics},$$

The MSE loss function is defined as (17) and (18), where *N* is the number of molecules, $p^{p}$ is the true molecular physicochemical properties, and $\beta_{1}\;\mathrm{and}\;\beta_{2}$ are learnable weight vectors.

### 4.3. Experiment Settings

We constructed an experimental environment using the Python language (version 3.8.20) and the PyTorch framework (version 2.4.1), configured on a Windows operating system. Our hardware platform includes an Intel(R) Core (TM) i9-14900HX (2.20 GHz) with 48 GB RAM.

We employed a 5-layer GIN model, which ultimately produces a 512-dimensional molecular structure representation. The initial node type features are 120-dimensional, comprising 118 atom types, 1 motif node type, and 1 super node type. The initial node degree is 11-dimensional. The edge type features are 7-dimensional, corresponding to single bond, double bond, triple bond, aromatic bond, self-loop, atom-to-motif edge, and motif-to-super-node edge. The edge ring features are 3-dimensional. ${MLP}_{link},\;{MLP}_{edge\_type},\;{MLP}_{node\_type},\;{MLP}_{structure}$ and ${MLP}_{fusion}$ are 2-layer MLPs with ReLU activation functions. ${MLP}_{node\_count}\;and\;{MLP}_{edge\_count}$ are 2-layer MLPs with Softplus activation functions. ${MLP}_{characteristics}$ consists of five stacked 2-layer MLPs with a ReLU activation function.

The fine-tuning strategy contains three steps in the downstream prediction tasks. Step 1: In order to learn optimal molecular structure representations, the GIN model and the structure representation predictor ${MLP}_{structure}$ are jointly fine-tuned in an end-to-end fashion. Step 2: To obtain the final optimized selected characteristics, the molecular stacking predictor ${MLP}_{characteristics}$ is fine-tuned independently in an end-to-end fashion. Step 3: In order to predict the physicochemical properties of the molecules, the parameters obtained from steps 1 and 2 are used as the initial parameters. Then, the GIN model is frozen, and the structure representation predictor ${MLP}_{structure}$, molecular stacking predictor ${MLP}_{characteristics}$, and fusion predictor ${MLP}_{fusion}$ are jointly fine-tuned in an end-to-end fashion.

### 4.4. Parameter Selection

**Figure 6:**
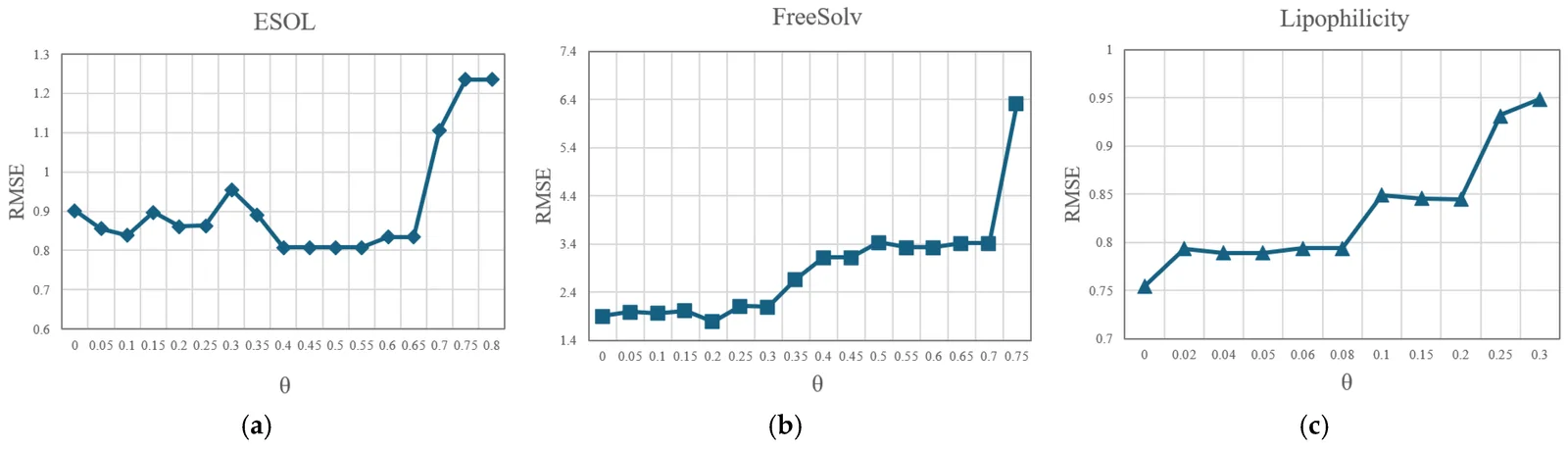
Dependence of RMSE on the correlation threshold parameter (*θ*) for three public datasets: (**a**) ESOL; (**b**) FreeSolv; (**c**) Lipophilicity.

We investigated the impact of model parameters on performance. Specifically, the correlation threshold *θ* in the molecular characteristic selection module determines the number of selected characteristics. Parameter analysis of the correlation threshold *θ* was conducted on the three datasets, with results shown in Figure 6. As shown in Figure 6, the optimal correlation threshold *θ* values for the three datasets are determined to be 0.4, 0.2, and 0.0, respectively. The final molecular characteristics selected for the three datasets are listed in Table S1.

## 5. Conclusions

In this study, a novel method is proposed to enhance the performance of molecular physicochemical property prediction, which effectively utilizes molecular structures and characteristics. The use of the molecule structure extraction model and the strategy of fusing molecular structure and characteristics significantly improved prediction performance. Experimental results on three widely used molecular property datasets demonstrate that the PMPP method surpasses existing competing methods. The proposed method has potential scientific applications, such as drug design and precision medicine.

## Figures and Tables

**Table 1:** Test performance of different methods on three datasets.

Methods	ESOL	FreeSolv	Lipophilicity
GROVER	1.350 ± 0.008	3.490 ± 0.083	0.757 ± 0.005
MGSSL	1.343 ± 0.012	2.986 ± 0.089	0.748 ± 0.005
MolCLR	1.113 ± 0.023	2.301 ± 0.247	0.736 ± 0.014
KANO	0.805 ± 0.021	2.001 ± 0.134	0.708 ± 0.007
HiMol	0.833 ± 0.001	2.283 ± 0.002	0.708 ± 0.002
Mol-AE	0.791 ± 0.020	1.994 ± 0.852	0.680 ± 0.148
PMPP (ours)	**0.7** **72 ± 0.009**	**1.** **874 ± 0.016**	**0.6** **53 ± 0.025**

Best performing methods for each dataset are marked in bold. Means and standard deviations of test RMSE are reported.

**Table 2:** The results of ablation studies on the three datasets.

Methods	ESOL		FreeSolv		Lipophilicity	
	RMSE	PCC	RMSE	PCC	RMSE	PCC
PMPP-w-S	0.903	0.908	2.371	0.776	0.643	0.730
PMPP-w-CS	1.255	0.838	2.820	0.790	0.791	0.669
PMPP-w-CI	0.902	0.907	1.900	0.870	0.854	0.649
PMPP-w-CO	0.807	0.926	1.792	0.875	0.755	0.820
PMPP-w-MACCS	1.472	0.764	1.923	0.850	0.940	0.563
PMPP-w-Morgan	1.658	0.636	3.651	0.479	0.937	0.569
PMPP	**0.7** **60**	**0.93** **5**	**1.8** **54**	**0.871**	**0.615**	**0.899**

Best performing methods for each dataset are marked in bold.

**Table 3:** Five compounds with the largest error reduction achieved by PMPP over the structure-only baseline method in ESOL.

Compound ID	Smiles	Label	PMPP	PMPP-w-S	LogP	Hydrophobe	Ring
Amitraz	CN(C=Nc1ccc(C)cc1C)C=Nc2ccc(C)cc2C	−5.47	−5.3201	−3.8993	4.8719	4	2
P,P’-DDE	ClC(Cl)=C(c1ccc(Cl)cc1)c2ccc(Cl)cc2	−6.9	−6.8258	−5.4174	6.1879	6	2
Etofenprox	CCOc1ccc(cc1)C(C)(C)COCc3cccc(Oc2ccccc2)c3	−8.6	−7.1885	−5.7910	6.3720	3	3
Triamterene	Nc3nc(N)c2nc(c1ccccc1)c(N)nc2n3	−2.404	−2.7723	−4.1478	0.8334	1	3
Etoposide	COc1cc(cc(OC)c1O)C6C2C(COC2=O)C(OC4OC3COC(C)OC3C(O)C4O)c7cc5OCOc5cc67	−3.571	−3.3275	−5.1878	1.3386	6	7

## Data Availability

All related data in this paper are public. The ZINC dataset for molecular structure representation can be downloaded from https://github.com/zaixizhang/MGSSL/tree/main/motif_based_pretrain/data/zinc (accessed on 16 April 2025) as described in MGSSL30. All three public molecular physicochemical datasets—ESOL, FreeSolv, and Lipophilicity—can be downloaded from the MoleculeNet website at https://github.com/deepchem/deepchem/tree/master/deepchem/molnet/load_function (accessed on 17 April 2025). The GuacaMol database can be downloaded from https://github.com/BenevolentAI/guacamol (accessed on 23 May 2024).

## Supplementary Materials

The following supporting information can be downloaded at https://doi.org/10.6084/m9.figshare.34911348: Figure S1 presents the t-SNE visualization of the structure–characteristics representation learned by PMPP on the Lipophilicity dataset. The Pearson correlations of each dimension of the MACCS and Morgan fingerprints with LogP on the GuacaMol dataset are shown in Figures S2 and S3, respectively. The final molecular characteristics selected for the three datasets are listed in Table S1. Each dimension of the MACCS and Morgan fingerprints with LogP on the GuacaMol dataset is provided in Tables S2 and S3, respectively. The description of each dim of MACCS fingerprints is included in Table S2_Description. The description of each non-zero value in Morgan fingerprints is provided in Table S4. The numerical values of the molecular descriptors in the ESOL, FreeSolv and Lipophilicity datasets are provided in Table S5.

## Abbreviations

The following abbreviations are used in this manuscript:

ADMET	Absorption, Distribution, Metabolism, Excretion, and Toxicity
LogP	The octanol–water partition coefficient
RMSE	Root Mean Square Error
PCC	Pearson Correlation Coefficient
HBDs	Hydrogen Bond Donors
HBAs	Hydrogen Bond Acceptors
PIMs	Positively Ionizable Moieties
NIMs	Negatively Ionizable Moieties
ZBGs	Zinc-Binding Groups
ARs	Aromatic Rings
HAs	Hydrophobic Atoms
HGs	Hydrophobic Groups
Mol_weight	Molecular weight
Hv_weight	Heavy atom weight
Hv_count	Number of heavy atoms
TPSA	Topological Polar Surface Area
SA	Synthetic Accessibility
QED	Quantitative Estimate of Drug-likeness
